# Using the canadian egg ladder in children with food protein-induced enterocolitis syndrome: a case series

**DOI:** 10.1186/s13223-023-00843-x

**Published:** 2023-10-06

**Authors:** Linlei Ye, Tiffany Wong, Elana Lavine, Victoria E. Cook, Stephanie C. Erdle

**Affiliations:** 1 Department of Pediatrics, University of Alberta, Edmonton, AB Canada; 2 Division of Allergy, BC Children’s Hospital, Vancouver, BC Canada; 3 Department of Pediatrics, University of Toronto, Humber River Hospital, Toronto, ON Canada

**Keywords:** Food protein induced enterocolitis syndrome, Allergy, Food ladder, Egg ladder, Pediatrics, Hypersensitivity

## Abstract

**Background:**

Current management of food protein-induced enterocolitis syndrome (FPIES) involves strict avoidance of the offending food for 12–18 months, followed by oral food challenge (OFC) under physician supervision. OFCs are resource-intensive and there is a lack of a universal standardized protocol for FPIES. Prolonged avoidance may increase the risk of IgE-mediated allergy, particularly in atopic patients. Food ladders have shown success in promoting accelerated tolerance in patients with IgE-mediated allergy. Our case series evaluated the safety of use of the Canadian Egg Ladder in patients with mild-to-moderate FPIES to egg.

**Methods:**

From May 2020 to November 2021, patients with mild-to-moderate FPIES to egg, defined as no history of lethargy or intravenous fluid administration, were started on the Canadian Egg Ladder. Instructions for advancing up the ladder were identical to using the Canadian Egg Ladder in patients with IgE-mediated allergy. Patients were followed every 3–6 months, at which time information was collected regarding progression up the ladder, symptoms while on treatment and interventions required. Treating allergists completed a survey to capture baseline demographic characteristics and prior tolerance to egg. Descriptive statistics were analyzed using MS Excel.

**Results:**

Twenty-one patients with mild-to-moderate FPIES were started on the Canadian Egg Ladder. Median age at initiation of the ladder was 10 months (IQR, 9–11). Nineteen (90.5%) patients completed the ladder, tolerating a serving size amount of cooked egg, over a median duration of 7 month (IQR, 4–9 months). Four patients (19.0%) had mild symptoms including vomiting (9.5%), pallor (9.5%), belching (4.8%), irritability (4.8%) and small spit up (4.8%). In three of the four patients, symptoms were the result of accidental exposure to a higher step of the ladder. There were no reports of lethargy. No patients required health care presentation or intravenous fluid administration. No patients discontinued the ladder.

**Conclusions:**

The Canadian Egg Ladder can safely guide the dietary advancement of egg-containing foods in patients with mild-to-moderate FPIES to egg, without the need for prolonged avoidance and resource-intensive OFCs.

## Background and rationale

Acute food protein-induced enterocolitis syndrome (FPIES) is a non-IgE-mediated allergy characterized by delayed, repetitive vomiting 1–4 h after ingestion of the suspect food in the absence of classic IgE-mediated symptoms [1]. Profuse vomiting can be followed by delayed diarrhea and pallor. Most cases are mild-moderate, with a few episodes of intermittent vomiting, pallor, and mild lethargy which resolve at home. Severe cases may involve lethargy and hypotension requiring intravenous (IV) fluid support and hospitalization.

Traditional management of FPIES involves eliminating the trigger food from the diet, with subsequent reintroduction under physician supervision in the form of an oral food challenge (OFC), typically 12–18 months after the most recent reaction [1]. There is no consensus regarding OFC protocol for FPIES and practices vary greatly [2]. Moreover, OFCs are labour-intensive and costly for the healthcare system and patients [3]. Recent studies are evaluating alternative approaches to management, shifting away from invasive procedures and conserving resources [4].

Prolonged food avoidance could be harmful, particularly in atopic patients who are at increased risk of developing IgE-mediated food allergies [5]. Caubet et al. described children with FPIES, and also detectable specific IgE antibodies to the food that triggered FPIES, developing IgE-mediated allergy following strict allergen avoidance [6]. Early and regular consumption of allergenic foods significantly reduces the risk of developing allergies in high-risk infants compared to strict avoidance [5, 7] .

The Canadian Egg Ladder is used to guide families in introducing increasingly allergenic forms of egg to children with IgE-mediated egg allergy, starting with extensively heated products which most children with IgE-mediated allergy can consume without an allergic reaction. [8] Studies have reported that children with FPIES to egg were able to tolerate egg in baked form without severe adverse reaction, providing precedence for our hypothesis that patients with FPIES can be safely started on the Canadian Egg Ladder [9].

This is the first study to evaluate the safety of using the Canadian Egg Ladder in patients with mild-moderate FPIES to egg.

## Methods

From May 2020 to November 2021, pediatric patients under 5 years of age with mild-moderate FPIES to egg, were started on the Canadian Egg Ladder [8]. The diagnosis of acute FPIES was based on the 2017 American Academy of Allergy, Asthma and & Immunology international consensus guidelines [1]. All patients had two or more stereotypic reactions to hen’s egg. Patients with severe FPIES, defined as lethargy, hypotension, need for IV fluid rehydration or hospitalization were excluded.

Treating allergists completed a survey to capture baseline patient demographic characteristics and prior tolerance to egg. Our study protocol was identical to the Canadian Egg Ladder used to guide patients with IgE-mediated allergy to egg [8]. All patients were provided with a copy of the Canadian Egg Ladder that included detailed instructions on how to gradually progress up the ladder from Step 1 to 4. All patients were given a prescription for sublingual ondansetron, counselled on how to treat nausea/vomiting and when to present to emergency care. Patients were followed every 3–6 months, at which time information was collected regarding progression up the ladder, symptoms while on treatment and interventions required. During each visit, patients were counselled on how to move up or hold the ladder if they experienced symptoms [8].

Descriptive statistics were analyzed using Microsoft Excel.

## Results

### Patient characteristics

Table 1 shows baseline patient characteristics. Twenty-one infants, thirteen males (61.9%) and eight females (38.1%), were included. There were no patients with severe FPIES that were included. The median age at initial reaction was 6 months (IQR, 5–7 months). All patients had a subsequent adverse reaction. 95% of patients were high-risk for IgE-mediated food allergy, defined as having a personal history of atopy or a first-degree relative with an atopic condition [5]. A third of the patients had a first-degree family member with non-IgE-mediated food allergies including three patients (14.3%) with family history of FPIES.

**Table 1:** Baseline Characteristics Prior to Starting Canadian Egg Ladder

Patient No.	Sex	Age at Initial Reaction (months)	Risk Factor for IgE-Mediated Allergy*	Multi-food FPIES	Prior Tolerance to Ladder step 1 foods	Prior Tolerance to Ladder step 2 foods
1	Male	6	Yes	No	Yes	Never tried
2	Female	6	Yes	No	No	Never tried
3	Male	7	Yes	Yes - avocado	Yes	Never tried
4	Male	7	Yes	No	Yes	Yes
5	Male	8	No	No	Yes	Never tried
6	Male	6	Yes	No	Never tried	Never tried
7	Male	6	Yes	No	Never tried	No
8	Male	5	Yes	No	Yes	Never tried
9	Female	4	Yes	No	Never tried	Never tried
10	Female	7	Yes	No	Yes	Yes
11	Male	5	Yes	No	No	Never tried
12	Male	5	Yes	No	Never tried	Never tried
13	Male	6	Yes	No	Never tried	Never tried
14	Female	7	Yes	No	No	Never tried
15	Male	8	Yes	No	Yes	Never tried
16	Female	5	Yes	No	Yes	Never tried
17	Female	4	Yes	No	Yes	Never tried
18	Female	6	Yes	No	Never tried	Never tried
19	Male	5	Yes	No	Never tried	Never tried
20	Female	5	Yes	Yes - oat	Never tried	No
21	Male	6	Yes	No	Never tried	Never tried

*Risk factors for IgE mediated allergy include atopic dermatitis, allergic rhinitis, asthma, first degree family history of atopy (parents or sibling with eczema, allergic rhinitis, asthma, IgE-mediated food allergy), diagnosis of IgE-mediated food allergy

Eliciting doses for initial reactions ranged from ¼ teaspoon to greater than 2 tablespoons of cooked egg. A third of the patients (33.3%) described pallor, another third (33.3%) reported to have appeared tired but responsive, and two patients (9.5%) described diarrhea. Eighteen infants (85.7%) were observed at home, while three presented to emergency care, with one requiring sublingual ondansetron.

### Safety of using Canadian Egg Ladder [8]

Prior to starting the ladder, nine (42.9%) and two (9.5%) patients had previously tolerated Step 1 and Step 2 foods, respectively. Five patients (23.8%) had reacted to Step 1 or 2 foods, while seven patients (33.3%) had never been exposed to egg from any step of the ladder.

The median age at initiation of the ladder was 10 months (IQR, 9–11 months) (Table 2). Nineteen (90.5%) patients completed the egg ladder over a median duration of 7 month (IQR, 4–9 months). The remaining two patients are currently on step 2. Four patients (19.0%) had mild symptoms including vomiting (9.5%), pallor (9.5%), belching (4.8%), irritability (4.8%) and small spit up (4.8%). Of these, three patients had symptoms following accidental exposure to a higher step of the ladder and were instructed to return to the previously tolerated step, with respect to volume and form of egg. One patient had a reaction while adhering to the ladder protocol. This patient had never tried baked egg prior to starting the ladder and developed vomiting and pallor after consuming 2 teaspoons of baked egg. The reaction was managed with sublingual ondansetron at home. No patients required health care presentation or IV fluid administration. No patients discontinued the ladder.

**Table 2:** Summary of Patient Experiences with Canadian Egg Ladder

Patient No.	Age at Initiation of Ladder (in months)	Step started at	Duration on Ladder (months)	Reaction on Ladder	Intervention	Completed vs. Current Step
1	7	1	7	Step 4, pallor and fussy due to exposure to larger volume of food	Reduced volume of food given	Completed
2	9	1	Ongoing	No	None	Step 2
3	10	1	9	No	None	Completed
4	11	2	3	No	None	Completed
5	10	1	4	No	None	Completed
6	10	1	5	No	None	Completed
7	12	1	9	Step 3, belching and small spit up due to unintended exposure to Step 4 foods	Reviewed avoidance after accidental exposure to a higher step food	Completed
8	9	1	9	Step 1, vomiting due to unintended exposure to Step 4 foods	Reviewed avoidance after accidental exposure to a higher step food	Completed
9	13	1	3	No	None	Completed
10	11	2	5	No	None	Completed
11	27	1	Ongoing	No	None	Step 2
12	10	1	11	No	None	Completed
13	11	1	7	No	None	Completed
14	8	1	7	No	None	Completed
15	9	1	7	No	None	Completed
16	11	1	4	No	None	Completed
17	7	2	8	No	None	Completed
18	10	1	4	No	None	Completed
19	11	1	10	No	None	Completed
20	10	1	8	No	None	Completed
21	12	1	12	Step 1, vomiting and pallor	Paused the ladder for 3 months, then successfully reinitiated it	Completed

## Discussion

Our study demonstrated that the Canadian Egg Ladder can safely guide the advancement of egg-containing foods in patients with mild-to-moderate FPIES to egg. Most patients successfully completed the Canadian Egg Ladder, tolerating a serving size of cooked egg. No patients required healthcare presentation. Three of the four patients who experienced mild symptoms had accidental exposure to a higher step of the ladder, highlighting the importance of counselling on the hidden forms of egg in common foods. All four of these patients successfully completed the ladder. These promising results provide support for using food ladders, which may reduce the healthcare burden associated with OFCs and increase access to treatment for patients. Gradual introduction of egg-containing foods may also be safer than consuming large doses at once in OFCs. The rate of acute, adverse reactions requiring intervention for hospital-based OFCs has been reported to range from 10 to 41%, although these studies include patients with severe FPIES [6, 10–12]. Furthermore, reducing the need for strict dietary elimination may alleviate stress and improve quality of life for families [13].

The literature reports variable median age of acquiring tolerance to the FPIES trigger food ranging from 18 to 63 months [14–16]. Sopo et al. demonstrated earlier rate of resolution to cooked egg compared to raw egg and proposed a shorter duration between last reaction and OFC for cooked egg [9]. Similarly, participants regularly consumed cooked egg in this case series andthe median age of resolution was 17 months, which is earlier compared to the current literature. Furthermore, there were no severe adverse reactions to baked egg in both our case series and the study led by Sopo et al. .

Study limitations include recall bias; however, patients were followed every 3–6 months, limiting the reporting timeframe. Excluding patients with a history of severe FPIES reaction may have impacted participants’ tolerance and degree of reactions while undergoing treatment with the Canadian Egg Ladder. Lastly, results were based on a small sample size of participants from a single Canadian province and may not be generalizable to patients in other regions. Further evidence is required to support daily allergen exposure in the management of FPIES.

This novel approach encourages safe expansion of the diet, minimizes the duration and burden of food avoidance, eliminates the need for costly and time consuming OFCs, and may contribute to earlier delabelling of FPIES.

## Data Availability

All data generated or analyzed during this study are included in this published article.

## Abbreviations

FPIES: Food protein induced enterocolitis syndrome
IV: Intravenous
OFC: Oral food challenge
OIT: Oral immunotherapy

## References

[1] Nowak-Węgrzyn A, Chehade M, Groetch ME (2017). International consensus guidelines for the diagnosis and management of food protein–induced enterocolitis syndrome: executive summary—workgroup report of the adverse reactions to Foods Committee, American Academy of Allergy, Asthma & Immunology. J Allergy Clin Immunol.

[2] Nicolaides R, Bird JA, Cianferoni A, Brown-Whitehorn T, Nowak-Wegrzyn A (2021). Oral food challenge for FPIES in Practice—A survey: report from the Work Group on FPIES within the adverse reactions to Foods Committee, FAED IS, AAAAI. J Allergy Clin Immunol Pract.

[3] Fong A, Ahlstedt S, Golding MA, Protudjer JLP. The Economic Burden of Food Allergy: What We Know and What We Need to Learn. *Curr Treat Options Allergy*. Published online April 2022. 10.1007/s40521-022-00306-5 PMC9046535 35502316

[4] Pena L, Guffey D, Minard CG, Anvari S, Davis CM (2017). The role of intravenous access during oral food challenges in food protein-induced enterocolitis syndrome. Allergy Asthma Proc.

[5] Abrams EM, Orkin J, Cummings C, Blair B, Chan ES (2021). Dietary exposures and allergy prevention in high-risk infants. Paediatr Child Health.

[6] Caubet JC, Ford LS, Sickles L, et al. Clinical features and resolution of food protein-induced enterocolitis syndrome: 10-year experience. J Allergy Clin Immunol. 2014;134(2). 10.1016/j.jaci.2014.04.008. 10.1016/j.jaci.2014.04.008 24880634

[7] Perkin MR, Logan K, Bahnson HT (2019). Efficacy of the Enquiring about Tolerance (EAT) study among infants at high risk of developing food allergy. J Allergy Clin Immunol.

[8] Chomyn A, Chan ES, Yeung J, et al. Canadian food ladders for dietary advancement in children with IgE-mediated allergy to milk and/or egg. Allergy Asthma & Clinical Immunology. 2021;17(83). 10.1186/s13223-021-00583-w. 10.1186/s13223-021-00583-w PMC8340453 34353372

[9] Miceli Sopo S, Romano A, Bersani G (2019). Cooking influence in tolerance acquisition in egg-induced acute food protein enterocolitis syndrome. Allergol Immunopathol (Madr).

[10] Sopo SM, Giorgio V, Iacono I, Dello, Novembre E, Mori F, Onesimo R (2012). A multicentre retrospective study of 66 italian children with food protein-induced enterocolitis syndrome: different management for different phenotypes. Clin Experimental Allergy.

[11] Lee E, Campbell DE, Barnes EH, Mehr SS (2017). Resolution of acute food protein-induced enterocolitis syndrome in children. J Allergy Clin Immunol Pract.

[12] Wang KY, Lee J, Cianferoni A (2019). Food Protein–Induced Enterocolitis Syndrome Food Challenges: experience from a large Referral Center. J Allergy Clin Immunol Pract.

[13] Meyer R, Godwin H, Dziubak R (2017). The impact on quality of life on families of children on an elimination diet for non-immunoglobulin E mediated gastrointestinal food allergies. World Allergy Organization Journal.

[14] Prattico C, Mulé P, Ben-Shoshan M (2023). A systematic review of Food Protein-Induced Enterocolitis Syndrome. Int Arch Allergy Immunol Published Online March.

[15] Vazquez-Ortiz M, Machinena A, Dominguez O (2017). Food protein–induced enterocolitis syndrome to fish and egg usually resolves by age 5 years in spanish children. J Allergy Clin Immunol Pract.

[16] Watanabe Y, Sakai H, Nihei M, Miura K, Kumaki S (2021). Early tolerance acquisition in hen’s egg yolk–associated food protein–induced enterocolitis syndrome. J Allergy Clin Immunol Pract.

